# Recycled Poly(Ethylene Terephthalate) from Waste Textiles with Improved Thermal and Rheological Properties by Chain Extension

**DOI:** 10.3390/polym14030510

**Published:** 2022-01-27

**Authors:** Wen-Jun Wu, Xiao-Li Sun, Qinghua Chen, Qingrong Qian

**Affiliations:** 1 College of Environmental Science and Engineering, Fujian Normal University, Fuzhou 350007, China; 17805931286@163.com (W.-J.W.); qrqian@fjnu.edu.cn (Q.Q.); 2 Engineering Research Center of Polymer Green Recycling of Ministry of Education, Fujian Normal University, Fuzhou 350007, China

**Keywords:** waste textile, PET fibers, chain extender, mechanical recycling, rheological property

## Abstract

Annual production of textile fibers is continuing to rise and the substantial discharge of undegradable waste polyester fibers can cause serious environmental and even health problems. Thus, the recycling and reuse of recycled poly(ethylene terephthalate) from waste textiles (rPET-F) is highly desirable but still challenging. Here, five chain extenders with a different number of epoxy groups per molecules were used to blend with discarded PET fibers and improve its viscosity and quality loss in the recycling process. The molecule weight, thermal properties, rheological properties and macromolecular architecture of modified r-PET were investigated. It was found that all modified rPET-F samples show higher viscosities and better thermal properties. rPET-F modified by difunctional EXOP molecules show linear structure and improved rheological properties. rPET-F modified by polyfunctional commercial ADR and synthesized copolymers exhibit a long chain branched structure and better crystallization. This study reveals a deeper understanding of the chain extension and opens an avenue for the recycling of PET textiles.

## 1. Introduction

Polyester fabrics, with poly(ethylene terephthalate) (PET) as the main ingredient, are the largest variety of chemical fibers, accounting for more than 80% of total chemical fibers [1,2,3,4]. With the development of the textile industry, the amount of waste polyester generated every year continues to increase significantly [5,6,7,8]. PET is a non-renewable petroleum resource, and it is difficult to degrade naturally in the natural environment. A large amount of waste polyester fabrics not only causes a waste of resources, but it also leads to a greater impact on the environment [9,10,11,12,13]. Therefore, the recycling and reuse of waste polyester fabrics is highly desired [14,15].

However, compared with PET bottles and food packaging, recycling PET fibers through mechanical recycling is still a great challenge due to its poor processability. The main problem in the recycling process of discard PET fiber is the lower inherent viscosity and viscosity loss caused by thermal and hydrolytic degradation. In addition, textile products often contain many additives, e.g., pigments, dyes, dispersants, etc., which also limit the recycling and reuse of PET fibers. Therefore, reused PET fibers are normally applied in lower value uses, e.g., reinforced fibers and fillers. In 2018, textile recovery in China was only 15% [16]. The mechanical recycling process has been well established for PET bottles. Substantial research has been carried out to upcycle PET flakes, and the most commonly used method is to add a chain extender during melt processing, which provides a reasonable solution for the recycling of discarded PET fibers [17].

The chain extender is an additive containing at least two functional groups that can react with the groups of macromolecular segments to generate new covalent bonds, which produce polymers with high molecular weight and recovery properties. As far as PET is concerned, the commonly used chain extenders with functional groups that can react with PET end groups include oxazolines [18,19], pyromellitic anhydride [20,21,22,23,24], organic phosphites [25,26,27,28] and epoxides [29,30]. Among them, the epoxides chain extenders are extensive, economical and efficient, offering great application prospects. Bikiaris and Karayannidis found that the excess epoxy groups would react with the hydroxyl end groups and react with the new hydroxyl groups created by the bonding of epoxides and carboxyl groups [31]. Haralabakopoulos [30] used five commercial diepoxides as chain extenders to extend PET molecules and they found the modified PET exhibited higher intrinsic viscosities than the unprocessed PET. Moreover, it is proved that the cyclic diepoxides have better chain extension effects than diglycidyl ether.

Polyfunctional chain extenders could also enhance the processability of recycled polyesters. One of the most common representative commercial chain extenders is the Joncryl^®^ ADR series of BASF, with a number average functionality of f_n_ > 4, which are introduced in various polymers to enhance the rheological properties, melt strength and molecular weight [32]. Xiao li-ren et al. studied the effects of Joncryl^®^ ADR 4370s on the relative molecular mass, distribution, branching and gel structure of recycled PET [33]. Supawee Makkam and Wanlop Harnnarongchai studied the effect of Joncryl^®^ ADR 4380 on the improvement of molecular structure and elongation properties of rPET [34]. Additionally, all found that the modified PET has a long chain branched structure and increased molecular weight after modified by ADR, which can cause a significant change in its rheological properties and improving properties.

In order to achieve better recovery properties of rPET, a series of copolymers with epoxy functional groups were also synthesized [35,36,37,38]. Benvenuta Tapia et al. synthesized a series of copolymers, e.g., a triblock polymers with middle block of poly(styrene-acrylonitrile) and two end blocks of poly(styrene-glycidyl methacrylate) (SGMA-SAN-SGMA), a triblock polymer with a middle block of poly(butyl acrylate) and extreme blocks of poly(styrene-glycidyl methacrylate) (SGMA-BA-SGMA) and a random copolymer of glycidyl methacrylate and styrene (GMS-*ran*-S) as a chain extender for polyester via reversible addition-fragmentation transfer (RAFT) and nitroxide mediated polymerization (NMP), respectively [39,40,41,42,43,44,45]. Moreover, the application of copolymers in the processing of polyester greatly improved the molecular weight and rheological properties of polyester.

However, the chain extension and its effect on the properties of waste PET fibers was rarely investigated. Herein, 1,4-Butanediol diglycidyl ether (EPOX) with 2 epoxy groups, Joncryl^®^ ADR-4468 with about 9–15 epoxy groups and three synthesized poly(glycidyl methacrylate-*co*-methyl methacrylate-*co*-styrene) (P(GMA-co-MMA-co-S)) with 38–132 epoxy groups per molecule, were used as a chain extender of discarded PET fibers. In this study, we present a comprehensive analysis of these five chain extenders with a different number of epoxy groups in order to obtain a deeper understanding of the influence on the molecule weight, molecular architecture and thermal properties of r-PET fibers. This study opens an avenue for the recycling of PET textiles.

## 2. Materials and Methods

### 2.1. Materials

Waste PET fibers from polyester textile wastes (referred to as rPET-F) and waste PET bottle flakes from discarded drink bottles (referred to as rPET-B) were supplied by Fujian Baichuan Resources Recycling Science and Company, Quanzhou, Fujian Province, China. rPET-F were exposed to a granulator to produce densified particles before use. Fiber-grade virgin PET particles (vPET) were purchased from DuPont Company, Wilmington, Delaware, USA. 2,2-Azobis(isobutyronitrile) (AIBN, initiator), glycidyl methacrylate (GMA, 99+%), methyl methacrylate (MMA, 99%) and styrene (St, 99%) were provided by Macklin, Shanghai, China. To remove the inhibitor and to obtain epichlorhydrin-free, GMA, MMA and St were passed through the basic alumina column. Tetrahydrofuran (THF, 99%), phenol and 1,1,2,2-tetrachloroethane were provided by Aladdin, Shanghai, China. The chain-transfer agent, S-1-odecyl-S-(α,α′-dimethyl-α″-acetic acid) trithiocarbonate (TC), was synthesized according to the literature [46]. EPOX with 2 epoxy functional groups was purchased from Aldrich and used as received. Joncryl^®^ ADR-4468, a styrene-acrylic multi-functional epoxide oligomeric agent with about 9–15 epoxy functional groups per molecule were purchased by BASF and used as received.

### 2.2. Synthesis of Copolymers

The block copolymers P(GMA-*co*-MMA-*co*-S) (PGMS) were synthesized via RAFT polymerization in a schlenk tube. The copolymers were prepared by the RAFT polymerization of GMA MMA and St. AIBN as the initiator, TC as the chain transfer agent and monomers and THF as the solvent were subsequently added to the tube. The solution was degassed using freeze-pump-thaw techniques (3 cycles) and the tube was immersed in an oil bath at 80 °C. Polymerization proceeded for 24 h under magnetic stirring and then stopped by cold water. The copolymer was purified by precipitation in methanol and filtration. The light-yellow powder was obtained after drying in a vacuum oven at room temperature for 2 days. PGMS with different content of GMA (defined as PGMS1, PGMS2 and PGMS3) were prepared by adjusting the feeding molar ratio of GMA/MMA/St from 9/2/1, 7/2/1 to 5/2/1.

### 2.3. Blending Process of PET with Chain Extenders

EPOX, Joncryl^®^ ADR-4468 and synthesized copolymers (PGMS1, PGMS2 and PGMS3) were used as the chain extender for rPET-F. The rPET-F was kept for 24 h in a 110 °C pre-heated oven before the blending process. Mixing of rPET-F with the chain extenders was performed in a RM-200B torque rheometer with a 50 mL internal chamber at 260 °C and 40 rpm for 7 min. 60 g of rPET-F and fixed amount of chain extender (0.5 wt%–1.5 wt% of rPET-F) were added in the intensive mixer. The melted samples after blending were cooled at room temperature.

### 2.4. The Melt Flow Rate

The melt flow rate (MFR) of PET samples were determined by a MFI452-A melt flow module, using an overhead weight of 2.16 kg at 257 °C. The tests of MFR were in accordance with GB/T 3682-2000.

### 2.5. Intrinsic Viscosity

The rPET-F, rPET-B, vPET and chain extended rPET-F were dissolved in 50/50 1,1,2,2-tetrachloroethane/phenol solution (*w*/*w*) and intrinsic viscosity (IV) values were measured by the Ubbelohde viscometer at 25 °C. The molecular weight was calculated in Equation (1) as follows [47]:

IV = 2.1 × 10^−4^ × (*Mn*)^0.82^ (1)

The insoluble content of the chain extended rPET-F were determined according to Xiao et al. [33].

### 2.6. Gel Permeation Chromatography

The molecular weights of the copolymers were monitored by gel permeation chromatography (GPC), using Agilent 1260 infinity Ⅱ equipped with a G7110B isocratic pump and G7162A refractive index detector at a temperature of 50 °C at a flow rate of 1 mL/min with polystyrene standards. The samples dissolved in DMF at room temperature and filtrated by 0.2μm PTFE filter prior to GPC test.

### 2.7. Nuclear Magnetic Resonance Spectroscopy

Proton nuclear magnetic resonance spectroscopy, ^1^H NMR (400.13 MHz), was performed on a Bruker 400 MHz spectrometer to measure the copolymer composition. About 10 mg of copolymer samples were dissolved in tritiated chloroform and scanned 32 times at room temperature.

### 2.8. Thermal Gravimetric Analysis

Thermal gravimetric analysis (TGA) was conducted in an analyzer (Q50, TA Instruments, New Castle, DE, USA) to measure the thermal stability of PLA. Approximately 8 mg of samples were heated from 30 to 600 °C with a heating ramp of 10 °C/min in an inert atmosphere (N_2_).

### 2.9. Differential Scanning Calorimetry

The melting and crystallization behaviors of the PET samples were recorded on a TA Instruments Q20 differential scanning calorimeter (DSC). The samples (6–8 mg by weight) were sealed in aluminum hermetic pans and subjected to a heat/cool/heat cycle over the temperature range 30–280 °C with a linear heating and cooling rate of 10 °C/min. The glass transition temperature (T_g_), crystallization and melting behavior of the polymer were determined from the second heating curve and analyzed using the commercially available Universal Analysis software (TA Instruments).

### 2.10. Dynamic Rheological Measurements

The complex viscosity and modulus were measured in a TA DHR-2 rheometer using standard 25 mm parallel plates. When the temperature stabilized at 265 °C for about 5 min, the sample was loaded between the parallel plates and melted at 265 °C for 1 min. Before each test, the parallel plate compressed the sample to a thickness of 1.00 mm. The stability of samples was checked through the dynamic time sweep test at 1 Hz and 265 °C with the stain amplitude of 1%. Frequency sweeps were performed in the range of 0.01–100 rad/s with a given strain amplitude of 1% within the linear viscoelastic region.

## 3. Results

### 3.1. Properties of the rPET-F, rPET-B and vPET

Compared with rPET-B, the recycling of rPET-F is still a challenge and rarely reported. Firstly, the rheological properties, thermal properties and chemical structure of rPET-B, rPET-F and vPET were investigated and compared. The IV value is an important index to measure the quality of polyester and it is also the most commonly used parameter to guide the actual production process. IV and MFR values of three PET samples are summarized in Table 1. The IV values of rPET-F is 0.58 dL/g, lower than rPET-B and vPET. At the same time, the MFR value obtained for rPET-F, rPET-B and vPET were 48.8 g/10 min, 18.6 g/10 min and 14.6 g/10 min. The lower IV value and higher MFR value of rPET-F implied that the decreased molecular weight and higher impurities during its use and recycling procedure [48].

**Figure 1:**
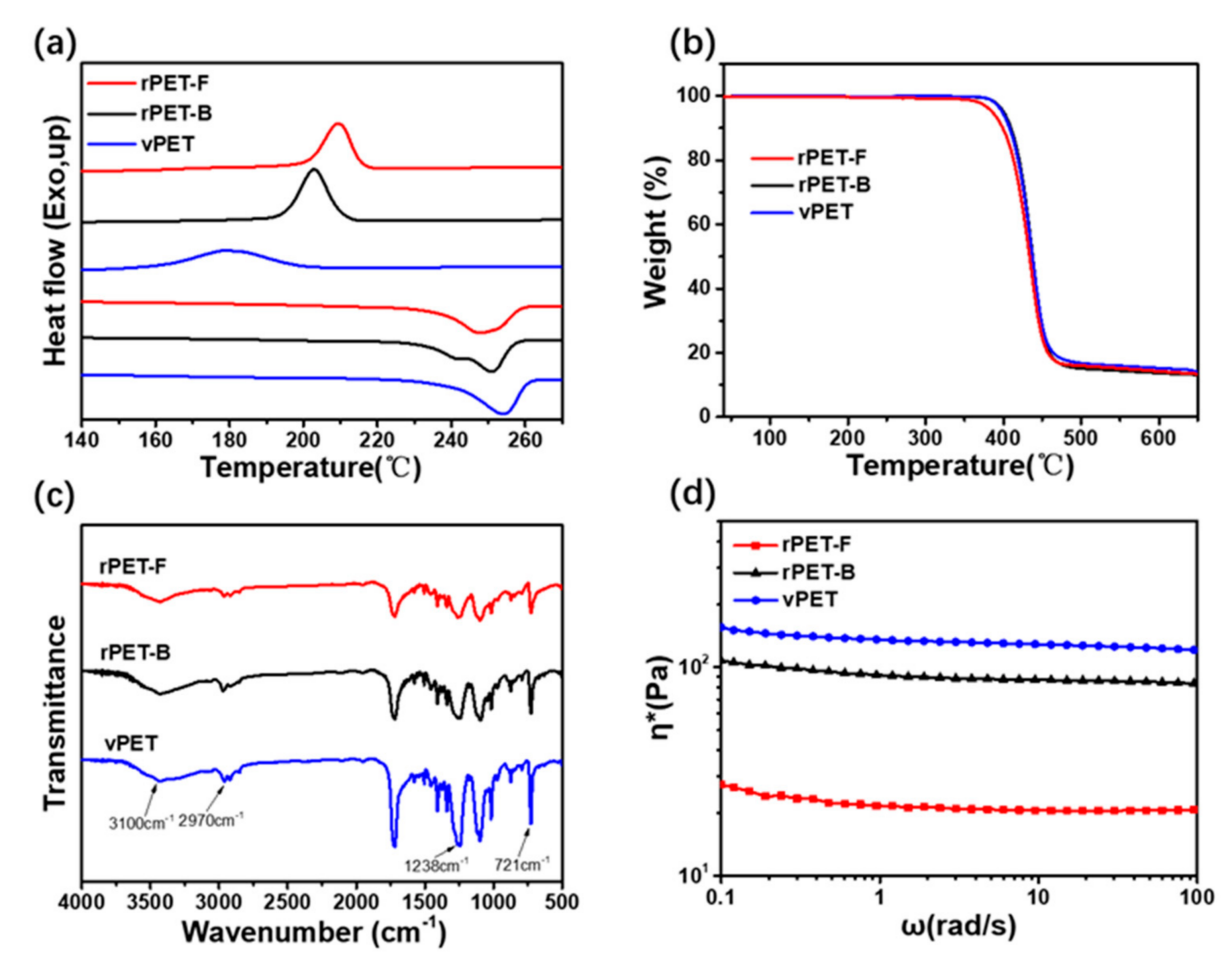
(**a**) DSC curves, (**b**)TGA curves, (**c**) FTIR spectra and (**d**) complex viscosity as function of frequency of rPET-F, rPET-B and vPET.

Table 1 and Figure 1a present the thermal behaviors. The thermal crystallization temperature (T_c_) of rPET-F is 209.4 °C, higher than that of rPET-B and vPET, which is attributed to the additives introduced during spinning (e.g., pigments, dyes, spinning oil, etc.). These impurities serve as nucleation sites to increase the crystallization temperature. In addition, the rPET-F shows a rather broad melting peak, which corresponds to the wide distribution of lamellae thickness caused by a wider molecular weight distribution [49]. Figure 1b displays the TGA trace of the rPET-F, rPET-B and vPET. As shown in Figure 1b and Table 1, the T_onset_ of rPET-B decreases 2 °C compared with vPET. It can also be seen that the T_onset_ of rPET-F reduces 14.8 °C in relation to vPET, which means the thermal stability rPET-F is worse than rPET-B and vPET. Such a result is related to the broken molecular chain mentioned above.

Figure 1c presents the FT-IR spectra of the PETs signals. The absorption peaks at 721 cm^−1^, 871 cm^−1^ and 1238 cm^−1^ indicate the presence of the aromatic ring. The absorption peaks at 3100 cm^−1^ and 3500 cm^−1^ are signed to hydroxyl groups (-OH). The peak of methylene oxy (-OCH_2_) appears at 1116 cm^−1^. One can observe that no significant difference in the infrared spectra of the three kinds of PET. This indicates that the chemical structure of the three PETs have basically not changed.

Rheological behavior can well quantify the changes in the molecular weight and structure of polymers. Figure 1d displays complex viscosity as a function of angular frequency for rPET-F, rPET-B and vPET. All the samples show the Newtonian behavior throughout the whole frequency range, which indicates linear chains [50]. It also can be seen that the rheological curve for rPET-F is characterized to be the lowest complex viscosity throughout the whole frequency range. Such behavior is associated with the results observed in the intrinsic viscosity, the melt flow rate and thermal properties, where the rPET-F present a lower molecular weight and more impurities than rPET-B and vPET.

To achieve the upcycling of rPET from waste textiles, the improvement of lower viscosity and worse thermal stability of rPET-F were highly needed. Here, the chain extension of rPET-F was designed to improve their properties. Additionally, the effect of chain extenders with different structure and functional group numbers on the property improvement were investigated in detail.

### 3.2. Synthesis and Characterization of Chain Extender

**Figure 2:**
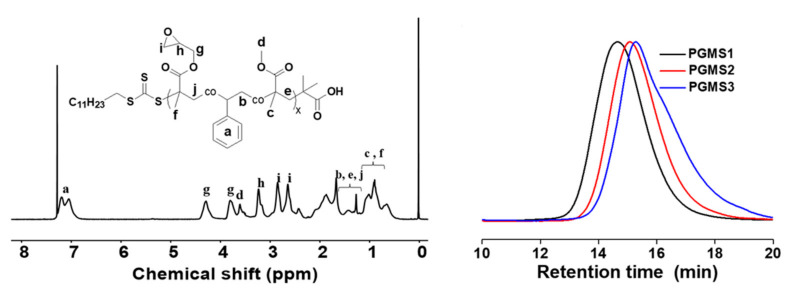
The ^1^H NMR spectrum of PGMS in CDCl_3_ (**left**) and the GPC curves of PGMS copolymers (**right**).

To investigate the influence of epoxy groups on the properties and structure of the modified rPET, EPOX, ADR-4468 and PGMSx, copolymers with different GMA content were chose as chain extenders. EPOX is a small molecule, which contains 2 epoxy groups capable of reacting with the carboxyl end of the PET segment (and to a lesser extent, the hydroxyl groups). Additionally, ADR-4468 has 5–9 epoxy groups and the number average molecular weight (*M_n_*) is –2000. In addition, RAFT polymerization was introduced to synthesis the copolymers PGMS with controlled molecular weight and chemical composition. Figure 2 left shows the ^1^H NMR spectrum for PGMS, which presents significant chemical shifts of the phenyl protons of St in the 6.8–7.4 ppm region and the methyl ester group of MMA at 3.6 ppm. Meanwhile, the chemical shifts of the methyl protons and methylene oxy (-OCH_2_) protons of GMA at 0.5–1.2 and 3.7–4.5 ppm, respectively. These results suggests that the PGMS copolymers are successfully synthesized.

To investigate the influence of epoxy group in PGMS on the structure of the modified PET, PGMSx with different GMA content, PGMS1 with 76.0%, PGMS2 with 70.4%, and PGMS3 with 60.6%, were synthesized. The GPC traces of PGMS1, PGMS2, and PGMS3 are shown in Figure 2 right. The GPC curves of PGMSx are unimodal and symmetric. Furthermore, Table 2 presents the number-average molecular weight (*M_n_*), weight-average molecular weight (*M_w_*) and polymer dispersity index (PDI) of PGMSx from GPC, revealing the *M_n_* in the range of 28,000–63,000 g/mol, the *M_w_* in the range of 51,000–99,000 g/mol and the PDI ranged from 1.58 to 1.80. Meanwhile, the contents of each segment of the PGMSx are listed in Table 2. The average number of epoxy groups per molecule (N_epoxy_) for PGMS1, PGMS2, and PGMS3 is 38, 70 and 152, respectively. Epoxide number of PGMS1, PGMS2, and PGMS3 is calculated as 0.24, 0.18, and 0.14 mol/100 g, which is lower than ADR (0.17–0.3 mol/100 g) and EXOP (0.99 mol/100 g). Multifunctional epoxide moieties in PGMS play important roles in the chain extension of PET.

### 3.3. IV and MFI of the Chain Extended PET Samples

The chain extension of rPET-F samples was performed in a torque rheometer and x% EPOX/rPET-F, x% ADR/rPET-F and x% PGMSx/rPET-F present the extended rPET-F samples with x% EPOX, ADR and PGMSx as chain extender, respectively. IV and MFR of chain extended rPET-F samples are shown in Table 3. As observed in Table 1, the MFR value of rPET-F is 48.8 g/10 min. It is obvious that with the addition of the chain extender, the MFR of all modified PET samples decrease. The higher the chain extender content is, the higher the MFR values of modified PET. After the chain was extended by EPOX, the MFR of 1.5%EPOX/rPET-F decreases to 24.2 g/10 min since EPOX have a slight effect on molecular weight in the specified blending time. It also could be found that the MFR of 1.5%ADR/rPET-F obviously decreases to 13.4 g/10 min, informing the successful reaction between ADR and rPET-F. Furthermore, the best result of MFR was obtained with the addition of 1.5% PGMS1, which decreases to 10.4 g/10 min. Compared with ADR-4468, PGMS contains more epoxide moieties per chain, which increases the molecular weight and more branched chains of the modified PET chain. In addition, for chain extended rPET-F modified by copolymers, 1.5%PGMS3/rPET-F shows a lower MFR value of 13.2 g/10 min due to the only 38 epoxy groups per chain. Therefore, the epoxy numbers per chain greatly influence the MFI values of modified PETs.

Due to the multifunctional groups of chain extenders, crosslinking structure are inevitably formed in the melting expansion process. It was found that rPET samples modified by PGMS are partly insoluble in the solvent during the IV measurements. Thus, an estimation of the crosslinking content of the insoluble samples was conducted and the results are shown in Table 3. It is found that both higher epoxy numbers per chain and higher content of the chain extender significantly increased the insoluble content of the sample.

### 3.4. Thermal Characterization of the PET Samples

**Figure 3:**
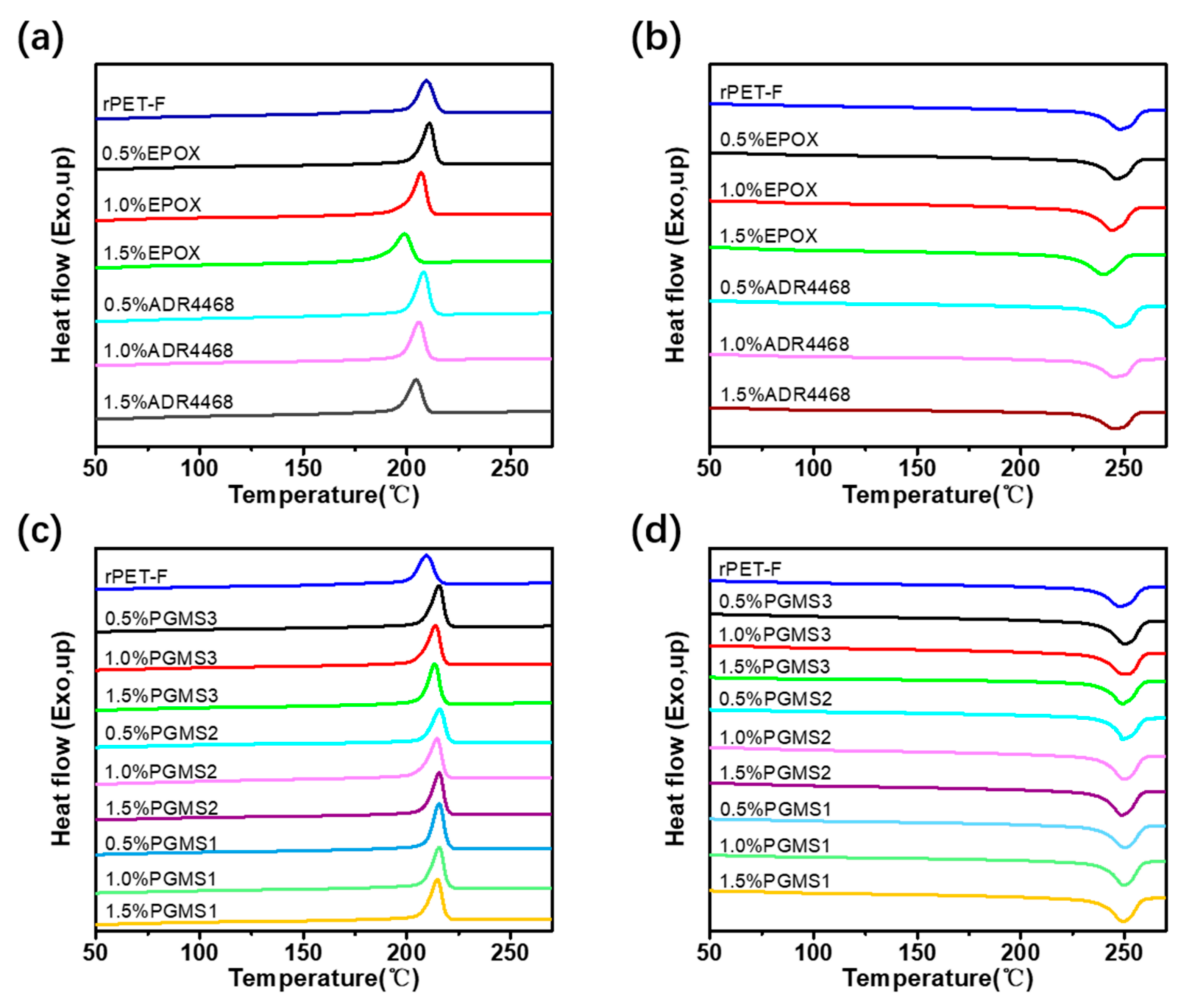
DSC curves for rPET-F modified by EPOX and ADR-4468, (**a**) cooling scan, (**b**) heating scan; DSC curves for rPET-F modified by different PGMS, (**c**) cooling scan, (**d**) heating scan.

The thermal behaviors of PET samples were investigated, and the results are shown in Figure 3 and Table 4. For the difunctional EPOX, the thermal crystallization temperature (T_c_) of 1.5%EPOX/rPET-F reduces to 198.7 °C, 10.7 °C lower than that of rPET-F. Moreover, the T_g_ of EPOX/rPET-F system decrease as increasing the content of EPOX. This result could be explained by the chain growth of rPET, which hinder the mobility of macromolecules and increase intermolecular entanglement. Such increased molecular entanglement reduces the crystallization rate, the degree of crystallinity, the crystallite size of the formed crystals and the amorphous regions, which increase segmental mobility leading to the lower T_g_ and T_c_.

The polyfunctional chain extenders, ADR-4468 and PGMSx, were also added as a chain extender to rPET-F. Interestingly, the results of PGMS/rPET-F and ADR/rPET-F exhibit completely opposite thermal behaviors and properties. As seen, T_c_ of PGMS/rPET-F and ADR/rPET-F do not decrease as EPOX/rPET-F. T_c_ of ADR/rPET-F is lower than that of rPET-F, which decreases from 208.0 °C to 204.5 °C with the increase in ADR addition. Additionally, all PGMS/rPET-F samples show higher T_c_ than rPET-F, ranged from 213.4 °C to 215.7 °C, which is less related with the content of PGMS. Compared with rPET-F, PGMS/rPET-F and ADR/rPET-F show a slight decrease in T_g_, from 80.1 °C to 77–79 °C. It has been demonstrated that the content of polyfunctional chain extenders within an appropriate value which did not suppress the crystallization as reported [51,52,53]. The addition of polyfunctional chain extender induces a branched structure, which restricts the movement of PET chains and suppresses the crystallization. Meanwhile, the branched sites could act as crystal nucleation regions which promote the crystallization of PET [51,54]. Moreover, it is interesting to note that the PS segment in the chain extender could also act as nucleating agents to promote crystallization, as previously reported [55]. In addition, the transition from linear to branched chains increases the number of free chain ends and disrupts the packing of the polymer chains, thereby reducing the impediment to segment mobility resulting in a decrease in T_g_. These things work together to generate minor changes in T_c_ and T_g_. The inhibiting effect is obvious for ADR and the positive effect is dominant for PGMS/rPET-F. Therefore, via tuning the chemical structure and N_epoxy_ of the chain extender molecule, the chain extended rPET-F samples with different thermal properties and macromolecular structures could be prepared for further applications.

### 3.5. Rheological Behavior of the PET Samples

**Figure 4:**
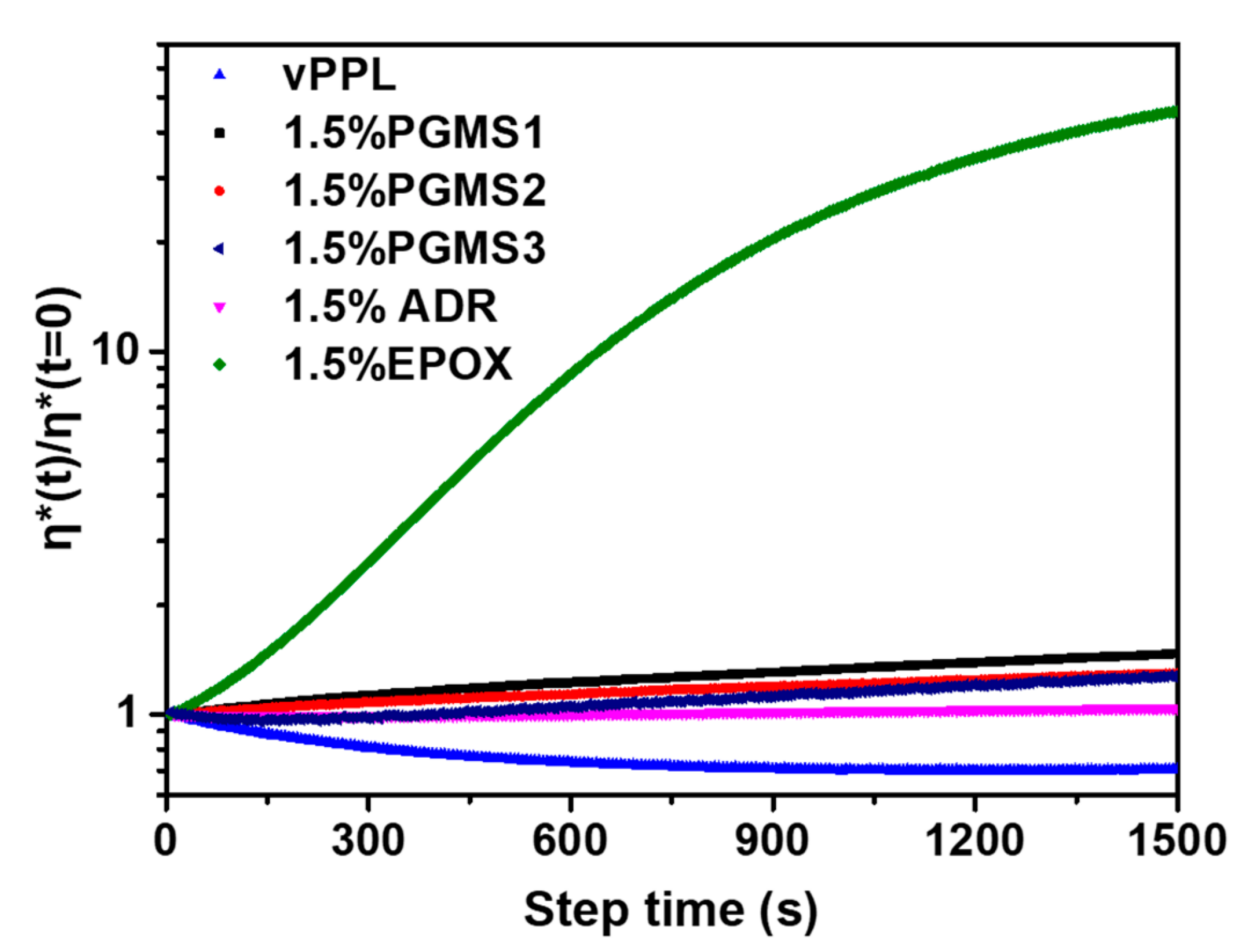
Dynamic time sweeps of various modified rPETs.

The dynamic rheological frequency sweep was conducted to investigate the structures of the chain extended rPET-F samples which were modified by different chain extenders. Figure 4 shows the dynamic time sweep of modified rPET-F for a time period of 30 min at 265 °C and a frequency of 6.28 rad/s, normalized by their initial values at *t* = 0. It is clear that the viscosity of rPET-F exhibits significant reductions with increasing test time, given that a loss in molecular weight caused by the accelerated chain-scission reactions at 265 °C.

The chain extension of rPET-F increases its complex viscosity as shown in Figure 4. Obviously, complex viscosity of extended rPET-F by 1.5% EPOX continues increasing from 39 Pa.s to 1800 Pa.s in the dynamic rheological frequency sweep, which indicates 1.5% EPOX/rPET-F presents an insufficient reaction in the torque rheometer, and EPOX needs a long time to react with rPET-F, even under high temperature and shear force, which is caused by higher epoxide number (0.99 mol/100 g for EPOX) and more epoxy group addition. To obtain the reliable rheological date of EPOX/rPET-F, the samples were annealed at 250 °C for 30 min to accelerate the chain-extension reaction. It should be noted that in the actual processing, the extrusion in the production process of factory could not provide sufficient time for reaction of rPET-F with EPOX. The residual epoxy group continues to react and influences the properties during use.

As shown in Figure 4, the complex viscosity of 1.5% ADR/rPET-F increases only 4% over 25 min, indicating the 1.5% ADR/rPET-F maintains a stable viscoelastic response. The slight increase in complex viscosity of 1.5% ADR/rPET-F also reflected that the ADR reacted fully with rPET-F in the torque rheometer. The PGMSx/rPET-F show roughly similar results to ADR/rPET-F, which demonstrated that the rPET-F have a rapid chain extension reaction with polyfunctional chain extenders.

**Figure 5:**
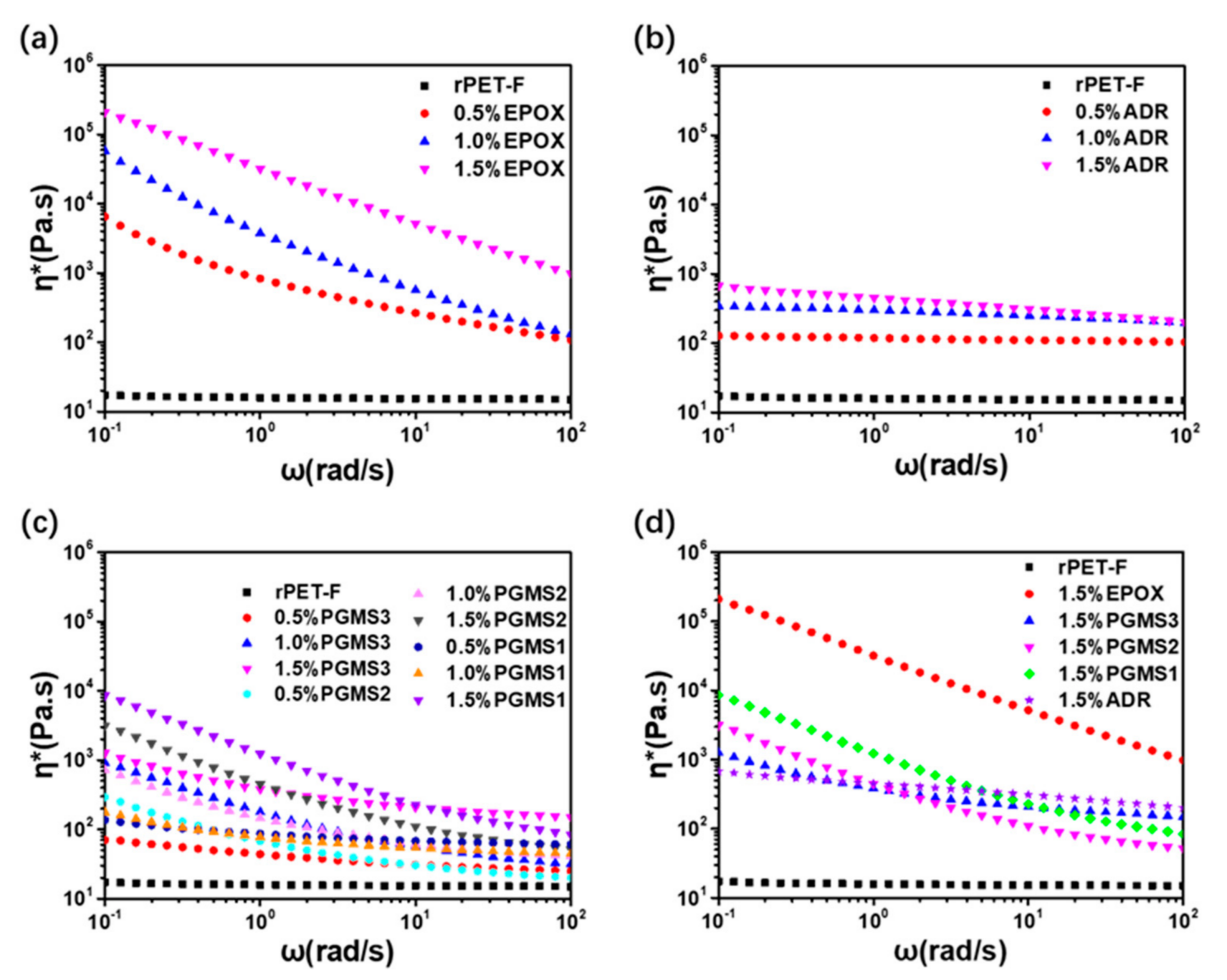
Complex viscosity (η*) vs. angular frequency (ω) for various modified PETs by (**a**) EPOX, (**b**) ADR-4468, (**c**) PGMSx and (**d**) different chain extender at in the same concentration.

In order to gain a clear idea of the final polymer topology, we conducted the dynamic strain sweep tests. Figure 5 shows the linear viscoelastic functions (η*) of chain extended rPET-F versus the frequency. It can be seen that rPET-F presents typical Newtonian behavior throughout the whole frequency range, which is characteristic of linear polymers. After 30 min annealing, EPOX/rPET-F samples show an obvious increase in the complex viscosity compared with rPET-F throughout the whole frequency region, which means the molecular chain growth. Meanwhile, for polyfunctional chain extenders, extended rPET-F shows about 5 to 300 times of complex viscosity higher than that of rPET-F in the low frequency area. Additionally, the complex viscosity of ADR/rPET-F and PGMSx/rPET-F gradually decreases with increasing frequency, which is a typical shear thinning behavior. Such behavior is due to the change in polymer chain entanglement and the enhancement of the relaxation mechanism [21,56].

In particular, 1.5% PGMS1/rPET-F presents the highest complex viscosity in the low frequency area and the highest shear sensitivity and non-Newtonian behavior as shown in Figure 5c,d, which is the result of the highest level long-chain branches (LCB) caused by higher N_epoxy_ [57]. At the same time, the increase in the shear thinning make processability more efficient at high rates.

The ADR/rPET-F exhibits a different shape of the viscosity functions, which shows a smaller decrease in complex viscosity with increasing frequency and higher complex viscosity system in a high frequency region compared to PGMSx/rPET-F. It was reported that the value of η* reflected the different branch structures of modified r-PET, especially at low frequencies [57]. Thus, the above results of ADR/rPET-F might be caused by the lower branched segments [58]. Thus, N_epoxy_ significantly influences the molecular architecture of the modified rPET-F. At the same time, insoluble chains in PGMSx/rPET-F also reveal that the highly branched structure and crosslinking structure was formed, which is consistent with the complex viscosity results.

**Figure 6:**
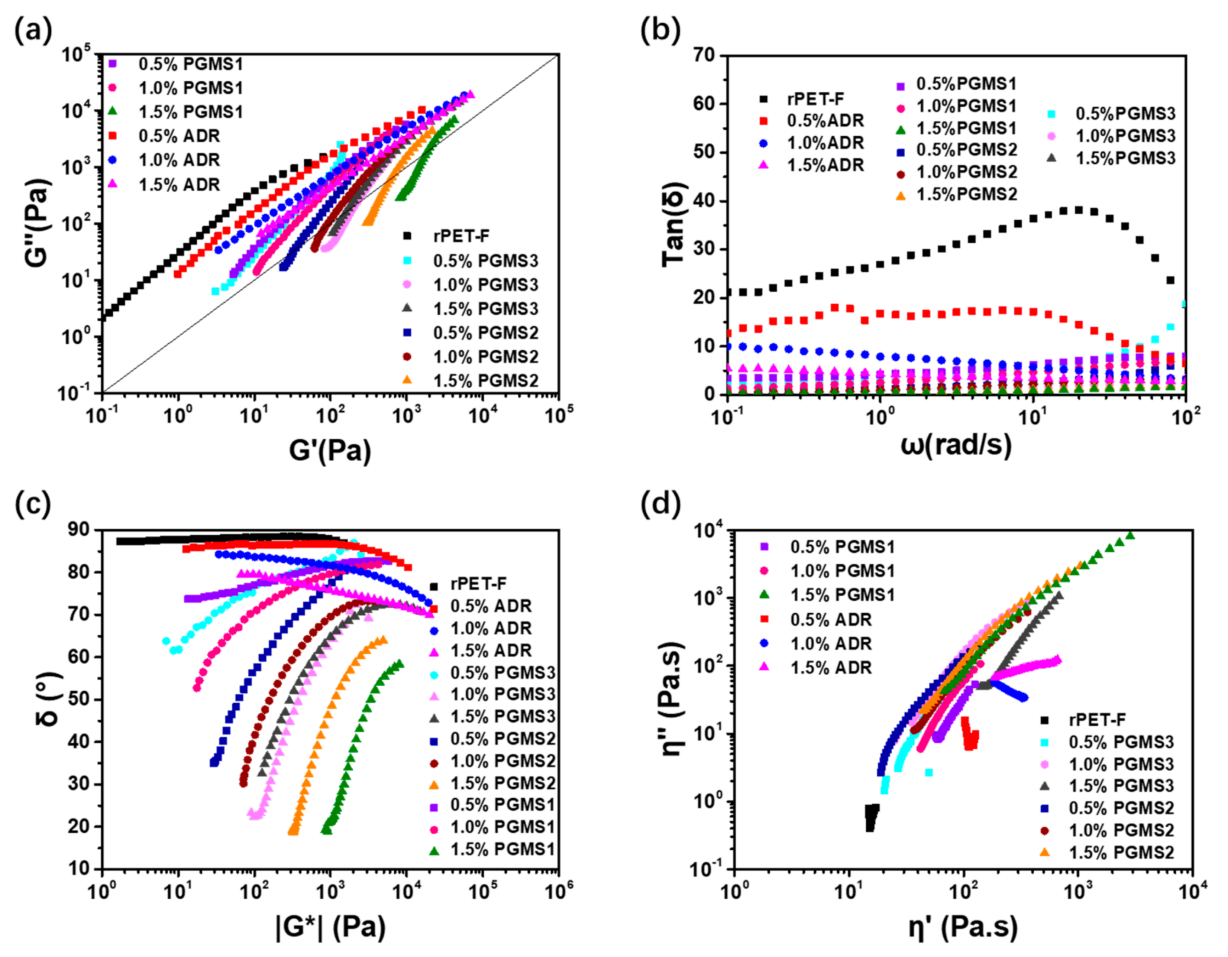
Han plots for various modified PETs (**a**), dynamic loss tangent (tan δ) as a function of ω for various modified PETs (**b**), vGP plots for various modified PETs (**c**) and Cole–Cole plots for various modified PETs (**d**).

Figure 6a shows the Han plots of rPET-F and modified rPET-F, the loss modulus (G″) as a function of storage modulus (G′). It can be seen that the curves are shifting toward to right with the increase in the amount of chain extender and number of epoxy groups on the chain extender molecule, which demonstrates that the extent of the G′ change is slightly higher than that of the G″ change with the frequency. The increase in G′ reveals that the behavior of modified PET by polyfunctional chain extenders transformed from liquid-like to solid-like with better melt elasticity. It is well known that melt elasticity is directly related to melt strength, which means that the chain extender improves the melt strength of the r-PET [39]. Figure 6b presents the damping factor (tan δ) of all samples, which referred to the angle that the strain lagged behind the stress. It is obvious that tan δ of rPET-F is much higher than that of modified rPET-F by polyfunctional chain extenders, which demonstrated that rPET-F exhibit more viscous than elastic. On the other hand, the tan δ of modified rPET-F decrease with the increase in the amount of chain extender and number of epoxy groups present on the chain extender molecule, which ascribes to the formation of a more branched structure [58,59,60].

To further distinguish the differences in the molecular structure of rPET-F modified by different chain extenders, the van Gurp-Palmen (vGP) plot and the Cole-Cole plot were drawn. By plotting phase angle verse complex modulus, the van Gurp-Palmen (vGP) plot is frequently used to characterize the topology of polymers [61,62,63]. It was known from the literature that the lower phase angle indicates the higher level of LCB [64]. As shown in Figure 6c, the rPET-F exhibits the typical behavior of a linear polymer, with a plateau of 90° in the whole |G*| range. For the 0.5% ADR/rPET-F, the vGP plots shows the same trend as rPET-F, suggesting that ADR/rPET-F system with the 0.5% ADR-4468 is a linear polymer. Moreover, the 1.0% ADR/rPET-F and 1.5% ADR/rPET-F present a lower δ in the higher value interval, which indicates that the existence of LCB with high content of ADR-4468. The vGP plots of the PGMS/rPET-F system deviate from the curve of the rPET-F and moves to a lower phase angle position. Therefore, 1.5% PGMS/rPET-F presents the highest LCB degree.

Previous research [58,60,65] has shown that the Cole-Cole plots of a linear chain is semi-circular and the diameter of the semicircle become larger as the molecular weight increases. The Cole-Cole plots of r-PET samples are shown in Figure 6d, and it can be seen that the Cole-Cole plots deviate from the semi-circular shape and gradually rise in the low-frequency region, which is due to the rheological changes in the longer relaxation time in the low frequency region, which is caused by the appearance of long-branched structures [66,67,68]. The irregular shape of the semicircle is caused by the low viscosity of rPET-F. The radius of the PGMS/rPET-F curve gradually increases, and the upward warping trend becomes more obvious with the increase in the amount of chain extender and N_epoxy_, which demonstrates an increase in the long-branched structure. However, the radius of the Cole-Cole curve of the ADR/rPET-F increased without the upward warping trend, indicating a lower degree of branching. Based on the results above, it can be seen that more functional groups lead to a more branched chain of the modified rPET-F.

**Scheme 1:**
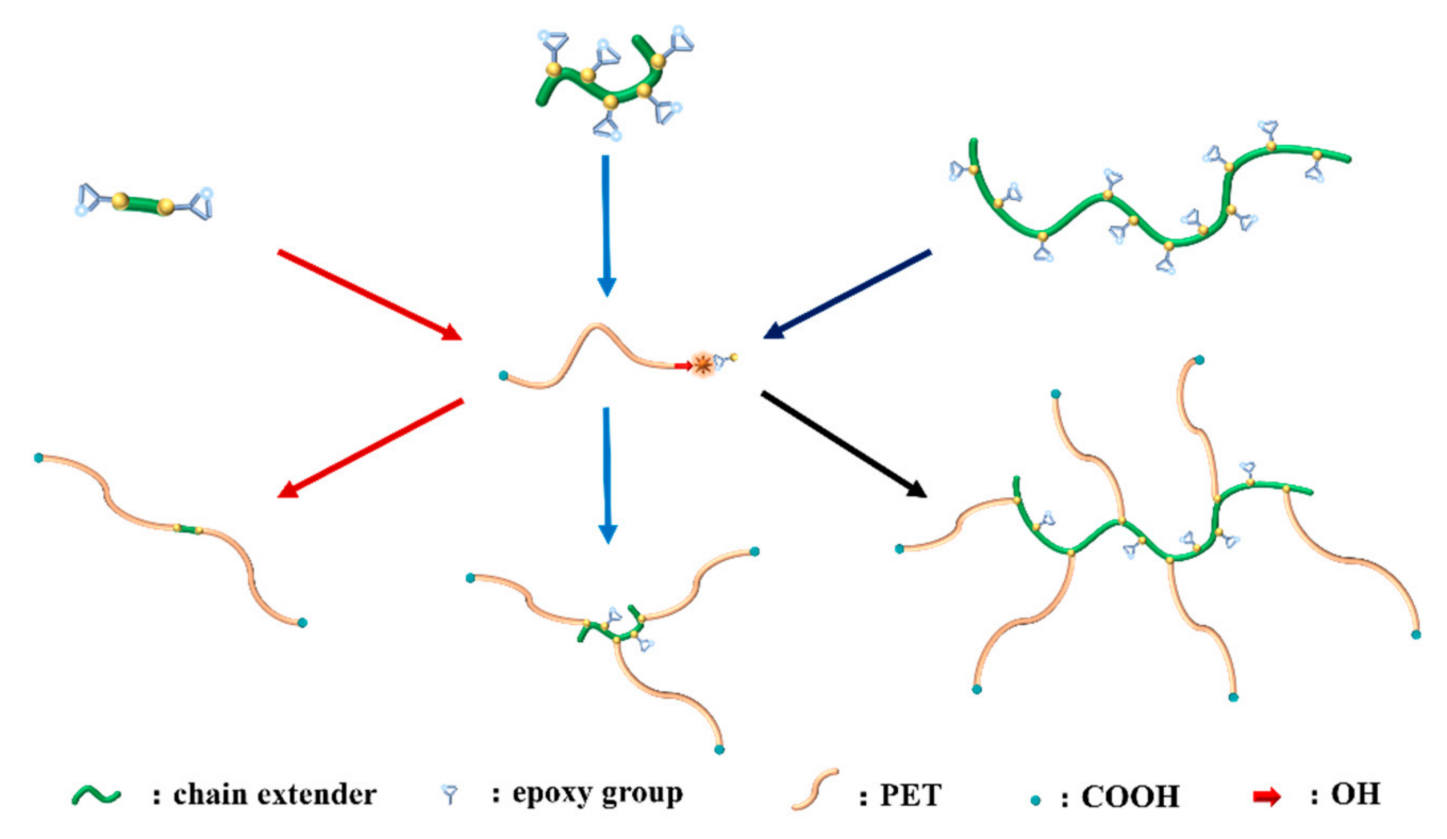
Schematic representation of chain extended rPET-F in this study.

According to the rheological behavior results of modified rPET-F, the average number of epoxy groups, as the paramount reaction site in chain extension, plays a pivotal role in the branch structures formation in modified rPET-F samples. Hence, branching is more likely to happen when ADR and copolymers are used with a high number of epoxy groups, as shown in Scheme 1. The above results show that the addition of chain extenders increases the complex viscosity of rPET-F. Moreover, the van Gurp-Palmen plot and Cole-Cole plot of modified rPET-F indicate higher levels of branching in the chain extender with more epoxy groups.

## 4. Conclusions

In this study, in order to achieve the upcycling of rPET-F, we synthesized a series of reactive copolymers PGMS with a high content of epoxy groups via RAFT polymerization as the chain extender of rPET-F, and compared this with EPOX and ADR-4468, which contain 2 and 5–9 epoxy groups, respectively. The effect of the number of epoxy groups on the thermal properties, rheological properties, and molecular structure changes of modified rPET-F were explored. Chain extension obviously improves MFR and IV values, and the crystallization temperature of EPOX/rPET-F and ADR-4468/rPET-F shifts to lower temperature, but PGMS addition leads to a higher crystallization rate compared to rPET-F. The rheological analysis shows that ADR-4468 and PGMS present faster reactive rates than EPOX, implying that ADR-4468 and PGMS possess better prospects in the actual production process in a factory setting. ADR-4468/rPET-F exhibit slight shear thinning behavior and a low degree of branching rheological results. On the other hand, the PGMS/rPET-F present more pronounced shear thinning behavior with higher LCB levels, resulting from more epoxy groups per chain. The study provides an effective strategy for the high-value utilization of rPET from waste textiles, suggesting that rPET-F with different qualities can be modified by chain extenders with a different number of epoxy groups, and can be used in a wide range of applications, such as spinning, film and foam materials, etc.

## Figures and Tables

**Table 1:** The IV, the MFR and thermal properties of PET samples.

Samples	IV (dL/g)	MFR (g/10 min)	T_g_ (°C)	T_m_ (°C)	T_c_ (°C)	T_onset_ (°C)
rPET-F	0.58	48.8	80.1	248.1	209.4	385.8
rPET-B	0.66	18.6	80.7	251.0	202.1	398.7
vPET	0.68	14.6	83.9	254.9	179.0	400.6

**Table 2:** Chemical composition, molecular weights, and PDIs of five chain extenders.

Samples	GMA (mol%)	S (mol%)	MMA (mol%)	N_epoxy_	*Mn* (g/mol)	*Mw* (g/mol)	Epoxide Number (mol/100 g)	PDI
PGMS1	76.0 ^1^	11.0 ^1^	13.0 ^1^	152	63,000 ^2^	99,000 ^2^	0.24	1.58 ^2^
PGMS2	70.4 ^1^	14.1 ^1^	15.5 ^1^	70	38,000 ^2^	61,000 ^2^	0.18	1.59 ^2^
PGMS3	60.6 ^1^	19.4 ^1^	20.0 ^1^	38	28,000 ^2^	51,000 ^2^	0.14	1.80 ^2^
ADR-4468	30–50	-	-	5–9	<2500	7250	0.17–0.3	>3
EPOX	-	-	-	2	202	-	0.99	-

^1^ Determined from NMR analysis. ^2^ Determined from GPC analysis.

**Table 3:** The IV and the MFR of chain extended rPET-F samples.

Samples	MFR (g/10 min)	IV (dL/g)	*Mn* (g/mol)	Insoluble Content (%)
0.5%EPOX/rPET-F	50.2	0.62	17,100	-
1.0%EPOX/rPET-F	44.6	0.66	18,400	-
1.5%EPOX/rPET-F	24.2	0.70	19,800	-
0.5%ADR/rPET-F	18.6	0.72	20,500	-
1.0%ADR/rPET-F	15.5	0.78	22,600	-
1.5%ADR/rPET-F	13.4	0.86	25,400	-
0.5%PGMS1/rPET-F	16.3	-		6.7
1.0%PGMS1/rPET-F	13.6	-		10.7
1.5%PGMS1/rPET-F	10.4	-		14.7
0.5%PGMS2/rPET-F	16.4	-		5.3
1.0%PGMS2/rPET-F	14.8	-		10.4
1.5%PGMS2/rPET-F	11.0	-		13.1
0.5%PGMS3/rPET-F	16.3	-		4.0
1.0%PGMS3/rPET-F	13.3	-		8.9
1.5%PGMS3/rPET-F	13.2	-		10.6

**Table 4:** The crystallization temperature, melting point and glass transition temperature of modified rPET-F blends.

Samples	T_c_ (°C)	T_m_ (°C)	T_g_ (°C)
0.5%EPOX/rPET-F	210.7	246.4	75.7
1.0%EPOX/rPET-F	206.9	244.5	73.7
1.5%EPOX/rPET-F	198.7	240.2	72.1
0.5%ADR/rPET-F	208.0	247.2	77.5
1.0%ADR/rPET-F	205.8	245.5	78.1
1.5%ADR/rPET-F	204.5	245.7	77.5
0.5%PGMS1/rPET-F	215.5	250.4	78.5
1.0%PGMS1/rPET-F	215.5	249.9	77.5
1.5%PGMS1/rPET-F	214.7	249.5	77.9
0.5%PGMS2/rPET-F	215.7	248.9	77.3
1.0%PGMS2/rPET-F	214.5	250.2	79.1
1.5%PGMS2/rPET-F	214.3	249.0	77.5
0.5%PGMS3/rPET-F	215.5	250.4	76.9
1.0%PGMS3/rPET-F	213.8	249.7	78.1
1.5%PGMS3/rPET-F	213.4	249.0	77.6

## Data Availability

The data presented in this study are available on request from the corresponding authors.
